# Invaders as Diluents of the Cercarial Dermatitis Etiological Agent

**DOI:** 10.3390/pathogens10060740

**Published:** 2021-06-11

**Authors:** Anna Stanicka, Łukasz Migdalski, Katarzyna Szopieray, Anna Cichy, Łukasz Jermacz, Paola Lombardo, Elżbieta Żbikowska

**Affiliations:** 1 Department of Invertebrate Zoology and Parasitology, Faculty of Biological and Veterinary Sciences, Nicolaus Copernicus University in Torun, 87-100 Torun, Poland; 293588@stud.umk.pl (Ł.M.); 285385@stud.umk.pl (K.S.); annacichy@umk.pl (A.C.); ezbikow@biol.umk.pl (E.Ż.); 2 Department of Ecology and Biogeography, Faculty of Biological and Veterinary Sciences, Nicolaus Copernicus University in Torun, 87-100 Torun, Poland; jermacz@umk.pl; 3 Limno Consulting, I-00124 Rome, Italy; p.lombardo@limnoconsulting.com

**Keywords:** dilution effect, *Trichobilharzia*, swimmer’s itch, alien species, feeding habits

## Abstract

Research on alien and invasive species focuses on the direct effects of invasion on native ecosystems, and the possible positive effects of their presence are most often overlooked. Our aim was to check the suitability of selected alien species (the snail *Physa acuta*, the bivalve *Dreissena polymorpha*, and the gammarid *Dikerogammarus villosus*) as diluents for infectious bird schistosome cercariae—the etiological factor of swimmer’s itch. It has been hypothesized that alien species with different feeding habits (scrapers, filterers and predators) that cohabit the aquatic environment with intermediate hosts of the schistosomatid trematodes are capable of feeding on their free-swimming stages—cercariae. In the laboratory conditions used, all experimental animals diluted the cercariae of bird schistosome. The most effective diluents were *P. acuta* and *D. villosus*. However, a wide discrepancy in the dilution of the cercariae between replicates was found for gammarids. The obtained results confirm the hypothesis that increased biodiversity, even when alien species are involved, creates the dilution effect of the free-living stages of parasites. Determining the best diluent for bird schistosome cercariae could greatly assist in the development of current bathing areas protection measures against swimmer’s itch.

## 1. Introduction

Widespread pulmonate gastropods are the first intermediate hosts of bird schistosomes (Trematoda: Schistosomatidae) and release cercariae infective for vertebrates that actively move in the water in search of the definitive host—waterfowl [1]. Moreover, cercariae can also attack accidental hosts, including humans, causing cercarial dermatitis ("swimmer’s itch") [2]. Swimmer’s itch is an emerging disease involving an intensely itchy rash but also general symptoms such as catarrh, diarrhoea, fever, insomnia [3,4,5] and in extraordinary cases disorders of the respiratory system and even anaphylactic shock [6]. Bird schistosome larvae may overcome the barrier of the mammalian skin and reach the internal organs [7]; more specifically, schistosomulae have been found in the lungs, heart, liver, kidney or intestine [7,8,9]. Some species of bird schistosomes can be extremely dangerous because they show high affinity to the central nervous system [10].

Cercarial dermatitis has been reported from nearly all continents, including Europe [11]. There are currently plentiful reports of outbreaks of swimmer’s itch from recreational water bodies [5,12,13,14,15,16]. Scientists are constantly looking for an effective method of protecting bathing areas from rashes of aquatic origin [17,18,19,20,21]. Methods that require human intervention related to the final host include (i.) reducing the population of bird hosts in recreational water areas by translocating these final hosts to other places, scaring them away using pyrotechnic harassment or even culling them and addling their eggs, as well as (ii.) the treatment of waterfowl with the anthelmintic drug praziquantel [22,23]. Bullard & Overstreet [24] consider that the elimination of the first intermediate host is the basal method of control for most digenean infections, which is carried out by introducing molluscivorous fishes [25], using molluscicides (e.g., CuSO_4_) [26], destroying the habitat of molluscs [17], or by manual and/or mechanical removal of molluscs from water bodies [23]. However, scientists postulate that these measures may be harmful to the environment and/or simply that their results are insufficient and short-lived [22,23,24].

In our latest work, we considered the possibility of breaking the life cycle of bird schistosomes at the stage of the first free-living larva—miracidium [20,27]. The transmission of free-living digenean larvae takes place in the presence of various communities of non-host organisms that may act on the dilution effect [28,29,30,31]. Non-host organisms can be a physical barrier in finding a host [32], or become dead-end hosts [33,34,35], as well as potentially being predators that feed on parasites during their free-living stages [36,37,38]. The last-mentioned possibility prompted us to use alien animals in our research, which are often very voracious and become food competitors of native species in the newly inhabited area [39]. Research on alien species, including invasive species, most often focuses on the negative effects of their presence in newly inhabited areas, while potential positive impacts are undervalued and overlooked [40]. It should be noted that the invaders can also influence the dynamics of native host–parasite interactions [20,41,42,43,44]. If the alien species is an incompatible host for local parasites, its co-occurrence in the various aforementioned ways leads to a dilution effect [20,35,42,45,46]. 

Our goal was to investigate whether common alien species with different feeding habits and frequently recorded in habitats with infectious bird schistosome larvae can dilute cercariae density by preying on them. We hypothesized that the overproduction of infectious parasite stages introduced into the environment enables the parasite to succeed in transmission to the next host, but the inclusion of new non-host species in the biocenosis may disturb this "balance". Therefore, we hypothesized that the cercariae of bird schistosome could be significantly diluted, becoming prey to newly co-inhabiting organisms.

## 2. Materials and Methods 

### 2.1. Bird Schistosome Cercariae Collecting and Counting

Six individuals of *Lymnaea stagnalis* (Linnaeus, 1758) (Gastropoda: Pulmonata: Lymnaeidae) infected with the *Trichobilharzia* sp. used in the experiment came from the natural environment—Lake Szymbarskie, Poland (53°36′52″ N, 19°30′39″ E). The lymnaeid snails were individually placed in beakers with 50 mL of dechlorinated tap water to obtain bird schistosome cercariae for the planned experimental setup. The shedding procedure was carried out in a breeding room with a constant temperature of 20 °C and natural light. After 22 h, the host snails were removed from the beakers and placed in an aerated aquarium where the snails were fed. After about a day, the snails were used again to release cercariae according to the described procedure. In total, each snail was used three times in as many days to obtain cercariae larvae. The long cercariae release time was used to obtain the highest density of the larvae to create optimal conditions for predators [21].

After removing the snails from the beakers, 1 mL of water with the larvae was collected from each of them three times with a micropipette. The contents of the beakers were gently hand-shaken before collection, and each of the three collections took place at a different height of the liquid—near the bottom, centre or surface. Each time the collected liquid was placed on a Sedgewick-Rafter chamber and poured with a small volume of 75% ethanol. Finally, the larvae in the chambers were accurately counted under a light microscope to estimate cercariae density.

### 2.2. Experimental Setup and Test Procedure

Three species of aquatic invertebrates were used as potential diluents of cercariae: the scraper *Physa acuta* Draparnaud, 1805 (Mollusca: Gastropoda: Physidae)—with 5 individuals (measuring 6.7 (SE 0.2) mm as shell length) per replicate beaker; the filterer *Dreissena polymorpha* (Pallas, 1771) (Mollusca: Bivalvia: Dreissenidae)—with 5 individuals (measuring 15 (SE 0.3) mm as shell length) per replicate, and the predator—*Dikerogammarus villosus* (Sowinsky, 1894) (Crustacea: Amphipoda: Gammaridae)—with 3 individuals (measuring 6.2 (SE 0.2) mm as total body length) per replicate. These molluscs and gammarids were collected from their non-native environment—Włocławek Dam Reservoir, Poland (52°37′04″ N, 19°19′29″ E). Each experimental diluent species was tested in 6 replicates. The replicates for a given alien species were set at the same time—each diluent species was used only once for testing. The experimental animals were not fed for two days before the experiment (according to the protocol presented by Selbach et al. [38] and Born-Torrijos et al. [47]). The experiment was carried out in the beakers described above, with the cercariae released into the water by the host snails. The use of clean beakers with no submerged structures that could have affected prey consumption by the differently feeding diluents allowed us to attribute any effect on cercariae density to active consumption by the tested alien species.

**Figure 1:**
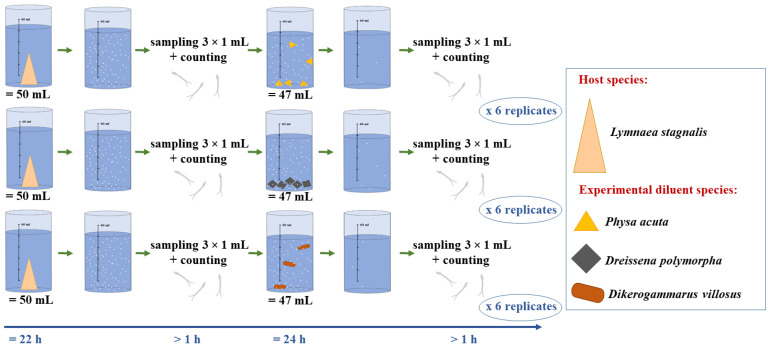
Schematic diagram of the experimental procedure.

About an hour (more precisely, the time during which the initial number of cercariae was counted) after taking the host snails (*L. stagnalis*) out of the beakers, the experimental invertebrates were added to the remaining 47 mL of water with the larvae. The potential cercariae diluents were kept in beakers with the cercariae for 24 h at a constant temperature of 20 °C and exposed to natural light. After this time, the diluents were removed from the beakers, and then under a stereoscopic microscope we checked whether any cercariae remained attached to their surface. Next, 1 mL of liquid was taken again three times from each beaker and the cercariae were counted on a Sedgewick-Rafter (General Oceanics, Miami, FL, USA) (Figure 1).

Following the results presented by Al-Jubury et al. [48], who showed that the cercariae of *Trichobilharzia* sp. remain active for up to 60 h at 20 °C, we assumed that the cercariae should be active throughout the experiments. However, if any cercariae died during the experiment and fell to the bottom of the beakers [49], then with the larval counting procedure described above, the fresh dead cercariae were also included in the counting.

Finally, the experimental animals were autopsied to check for the presence of digenean trematodes infection because they originated from the wild, and the presence of the infection could affect their feeding abilities [50]. To look for the presence of parasites, the experimental animals were crushed carefully and their soft parts were examined under a light microscope.

### 2.3. Data Analysis

The initial and final numbers of cercariae were calculated from 3 subsamples col-lected from each replication independently. To confirm the ability to consume cercariae by selected diluents we compared the initial and final cercariae densities (number of indi-viduals per mL) by means of paired sample t-tests, which can be applied regardless of data distribution type [51]. The analyses were carried out using SPSS 25.0 package (IBM Inc.).

## 3. Results

**Figure 2:**
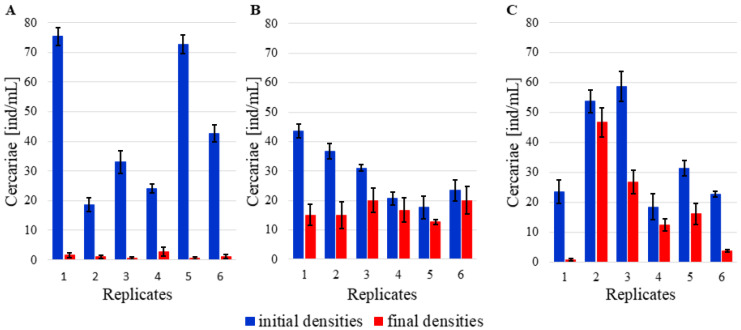
Bird schistosome cercariae in beakers (ind/mL, mean values ± SE, *n* = 3 for each tested replicate) before and after exposure to following experimental invertebrate species: (**A**) *Physa acuta* (5 specimens), (**B**) *Dreissena polymorpha* (5 specimens), (**C**) *Dikerogammarus villosus* (3 specimens).

**Figure 3:**
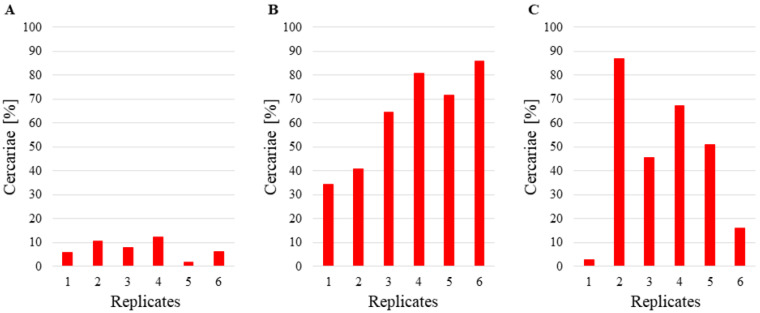
Average densities of bird schistosome cercariae remaining (%) after exposure to following experimental invertebrate species: (**A**) *Physa acuta* (5 specimens), (**B**) *Dreissena polymorpha* (5 specimens), (**C**) *Dikerogammarus villosus* (3 specimens).

Beakers with *P. acuta* had an initial average density of 18.7 to 75.3 cercariae per mL, depending on the tested replicate (Figure 2). After the experiment, an average of 1.8 to 12.5% cercariae remained in the beakers (Figure 3), and the difference between initial and final cercarial densities was statistically significant (t = 4.226, df = 5, *p* = 0.008).

Beakers with *D. polymorpha* initially contained 17.7 to 43.7 cercariae per mL (Figure 2). Carcariae density after the end of the experiment with *D. polymorpha* was 34.3 to 85.7% of initial density (Figure 3). The initial vs. final density difference was statistically significant (t = 2.842, df = 5, *p* = 0.036).

The beakers with *D. villosus* had an initial density of 18.3 to 58.7 cercariae per mL (Figure 2), and an average of 2.9 to 86.6% of the initial cercariae density had remained in the beakers (Figure 3). Again, the observed difference between the initial and final cercarial densities was statistically significant (t = 4.229, df = 5, *p* = 0.008).

Autopsy of the experimental animals revealed no natural infection with digenean trematodes. In the gills of about 40% of the investigated individuals of *D. polymorpha*, the presence of a single live cercaria of bird schistosome was recorded.

## 4. Discussion

The obtained results indirectly support the hypothesis that the increased biodiversity of the environment may disturb the transmission of the parasite to their host [28,34,52,53,54,55]. Non-host species may feed on free-living parasite cercariae [54,56], which is an important and underestimated factor influencing the dynamics of parasite infection [21]. Digenean cercariae can be a permanent component of the diet of non-host organisms, which allows for their long-term survival and even reproduction [57]. McKee et al. [58] showed that dragonfly larvae that consumed equivalent masses of either zooplankton (*Daphnia* spp.) or digenean cercariae grew equally well. Moreover, the cercariae may be selectively chosen by such organisms [59].

Many research studies [21,38,52,53,55,59,60] support our finding that potential consumers of free-living cercariae are species with different feeding modes—scrapers, filterers (which may ingest the cercariae only as a byproduct of their grazing on the substrate or water filtering) and active sensu stricto predators. Additionally, our results suggest that there is a difference in the intensity of the dilution effect by scrapers, filterers and predators. Hopper et al. [37] indicate that not all co-occurring non-host species are of equal importance, even within representatives of one feeding functional group. This point of view supports Welsh et al. [61] who suggest that parasite removal rates by predators are species specific, while Selbach et al. [38] emphasize that the use of cercariae as prey is highly dependent on the interspecific interaction between their dispersion behaviour and the feeding behaviour of predators. However, other factors influencing cercarial consumption have not been taken into account, such as diluter density or initial density of cercariae [21,53].

Selbach et al. [38] found a significant reduction in the number of cercariae only in the case of the bottom-dwelling *Coitocaecum parvum* (Digenea: Allocreadiidae), among the investigated cercariae with different behaviour as prey for *P. acuta*. Therefore, the feeding of *P. acuta* on cercariae may seem to be quite a controversial result, which mainly shows swimming and resting behaviour as well as concentrating below the water surface [1]. Given the lack of control experiments, we could expect lower real consumption, because of the sticky nature of schistosome cercariae, for example, which would allow cercariae to be “lost” before being counted. The cercariae of *Schistosoma japonicum* (Trematoda: Schistosomatidae) have a truly sticky nature, and adhere to even glass and plastic surfaces [62]. According to our personal observations, the cercariae of bird schistosomes exhibit similar adhesive properties to *S. japonicum*. The cercariae of bird schistosome attached to the walls of the beakers (or some solid surface in an aquatic environment) can be scraped off by grazing gastropods such as *P. acuta*. On the other hand, snails can slide upside down on the water surface [63], apparently "grazing" on floating fine matter (e.g., pollen or parasite propagules). It should be noted here that *P. acuta* is very active in the presence or in search of food [64]. Another possibility seems to be that the cercariae died during the test and their bodies were simply scraped off from the bottom of the beakers by grazing *P. acuta*. 

The sticky nature of bird schistosome cercariae may have contributed to a smaller dilution effect by *D. polymorpha* than by *P. acuta* under the experimental conditions, as *D. polymorpha* is considered to be an efficient filter feeder [65]. The cercarial dilution effect with *D. polymorpha* is not as spectacular as in the case of *P. acuta*, but taking into account the huge densities of dreissenid population achieved in the environment [66], it seems to us that *D. polymorpha* may play the role of an important dilution of the free-swimming larval stages in the natural system. Moreover, *D. polymorpha* also collects oocysts, cysts and spores of various species dangerous to humans [67,68,69,70,71]. In general, research about the use of different species of bivalve molluscs as pathogen collectors indicates the usefulness of these organisms for the dilution effect of parasite propagules [38,72,73,74,75,76]. However, it seems quite astonishing that the used filter feeder—*D. polymorpha*—which feed without reaching saturation [77], did not remove the greater number of cercariae. Géba et al. [71] showed that parasite oocysts can be bioaccumulated in the tissues of *D. polymorpha*, but can also be partially released via pseudofaeces. Gopko et al. [78] showed that the filter feeder *Anodonta anatina* (L.) (Bivalvia: Unionidae) effectively removed the furcocercariae of *Diplostomum pseudospathaceum* (Digenea: Diplostomatidae) from the water, but they suggest that the cercariae are probably transformed into pseudofeces and poorly ingested, similar to the studies presented by Bontes et al. [79] with the use of similar-sized filamentous cyanobacteria. We performed an autopsy of the experimental individuals of *D. polymorpha* which revealed the presence of live bird schistosome cercariae in the gills of several individuals. As a result, a question arises that needs further research as to whether cercariae can survive the journey through the body of filter feeders and whether individuals released via pseudofaeces are still infectious.

*Dikerogammarus villosus* is a highly effective predatory invasive amphipod. This gammarid can prey on crayfish eggs and hatchlings even directly from females’ abdomens [80]. Our results indicate that *D. villosus* may also prey on bird schistosome cercariae, although there are reports that this predator generally preys on larger prey in the environment [81], and the consumption of cercariae is strongly dependent on the size of both prey and predator [82]. Moreover, Welsh et al. [53] carried out an experiment on the dilution of cercariae using a very broad spectrum of non-host organisms and showed that amphipods may play a very important role in the dilution of the larvae. Additionally, Born-Torrijos et al. [47] showed that another species of widespread freshwater gammarids also efficiently consumes digenean furcocercariae, including genus Trichobilharzia. However, we have observed wide between-replicate variability in the dilution of the parasitic cercariae by *D. villosus*. During the experiment, the gammarids contaminated the water with their faeces, which made it a cloudy and slightly orange colour, which could affect their ability to see small prey (and eat all the prey in the beakers). Christensen et al. [83] and Schotthoefer et al. [56], who have used fish and copepods to dilute cercariae, also indicate that the ability of visual predators to eat the larvae is limited by cloudy water. Differences in cercariae consumption were found also for visual predators such as fish and larval damselflies observed under either light or dark conditions [82]. In addition, although all gammarids were starved for the same length of time prior to the experiment, we can not rule out the possibility that individual predators may have experienced different levels of hunger at the beginning and during the experiment. As suggested by Born-Torrijos et al., [47] the continuous movement of prey may likely facilitate a constant consumption by visual predators such as amphipods. Therefore, if the predators did not feed from the beginning of the experiment, with the duration of the experiment more and more cercariae may have become less attractive prey, because due to the length of our experiment and the short life span of the cercariae, the cercariae may have slowed down their movement and sunk later in the experiment.

Galaktionov & Dobrovolskij [84] emphasize that the production of cercariae is a key element in the success of digenean trematodes and Soldánová et al. [13] indicate that snails infected with bird schistosomes release huge amounts of infectious cercariae. We believe that reducing the number of bird schistosome cercariae in recreational waters could significantly decrease the risk of swimmer’s itch outbreaks. The dilution effect caused by feeding on free-living bird schistosome cercariae could be used to support chemical and mechanical means of protecting bathing areas against these parasites. Further research is needed to determine the best diluent, the presence of which should not be harmful to the local ecosystem. The diluent should also not become a source of other species of parasites. For example, *P. acuta*, on the one hand, is not considered to be a significant competitor for other snails [85], but on the other hand, in new areas, it plays the role of the second host of Echinostomatidae and Plagiorchiidae [86]. However, metacercariae abundance may [73] or may not be related to host densities [87]. These contradictory statements support the hypothesis put forward by Buck et al. [88] that every host–parasite interaction requires individual research and interpretation. From an ecological point of view, it seems interesting to investigate possible differences in feeding on the cercariae between native and alien species of scrapers, filterers and predators. The feeding behaviour of experimental animals in the presence of alternative prey will also be a significant aspect of the research, which we plan to implement soon.

## Data Availability

Raw and processed data will be shared on reasonable personal request directly from the corresponding author.
